# Homes in the Hills for Nursing Sisters

**Published:** 1887-12-03

**Authors:** Nora Roberts


### HOMES IN THE HILLS FOR NURSING SISTERS.

By the Lady Nora Roberts.

The absence of skilled nursing in the military hospitals in India has long been felt to be a serious evil by all those who have given the subject a thought, and constitutes a grave hindrance to the successful practice of military medicine in this country. Such a cruel want, I believe, could not have been allowed to continue so long unsupplied were it generally known that there is absolutely no system of nursing in the military hospitals, or were it possible to realize the number of lives that are annually lost among our soldiers (officers and men) for want of that skilled and careful attendance which is often the one thing needed to pull a patient through a dangerous illness, especially those protracted and serious cases of enteric fever, etc., to which the young men who nowadays come to India in such largely increased numbers are peculiarly subject. A military doctor has assured me that almost every medical officer in this country must be able to call to mind numerous instances where men's lives might have been saved had careful nursing been available; and many of them keenly deplore deaths which might have been averted had competent nurses been at hand.

During the last cold weather two young officers of the Leinster Regiment died, within a few days of each other, at Faizabad; two also died at Peshawur; and during the first and second weeks of this month fourteen men were lost at Peshawur alone from enteric fever, etc. Even as I write I hear of two young officers of the Royal Irish Fusiliers being dangerously ill of typhoid fever at Cherat. Both died a few days afterwards.

How sad to think of these young men, far away from home and friends, hopelessly ill, and unattended save by the rough and ignorant (however kindly) orderly detailed for attendance on them!

A great responsibility seems to me to rest upon all English men and women to do all they possibly can to supply this pressing need for nurses. I believe that an appeal would be heartily responded to by all those interested in the British soldier for help to raise a fund for the purpose of assisting to provide a staff of nursing sisters, whose services would be available wherever there might be a sickness among soldiers in peace time, and at the base hospital in time of war, and I believe that devoted women would be forthcoming in sufficient numbers for such work; but it is essentially necessary to the success of such an undertaking that the movement should be recognized and, to a certain extent helped by Government.

When one considers what an expensive article the British soldier is—costing the State, as he does, £100 on landing in India—it seems certain that, on the score of economy alone, altogether setting aside the humane aspect of the question, the Government would find it well worth its while to spend some money in providing him with the skilled nursing care which would in all probability save his life.

In England, where soldiers are not so costly, and are not nearly so subject to those protracted illnesses which require such unremitting and intelligent care, and where there is a trained body of men, the Medical Staff Corps, to look after sick soldiers, the nursing sister has been found to be a necessity, and is happily now a recognised institution in all the large military hospitals. Her good work in South Africa and Egypt has been gratefully acknowledged by the Home Government, as well as by those fortunate individuals who have been the objects of her care, and has been testified to by the medical officers, who feel how largely their success in difficult cases has been due to her devoted attention.

Surgeon-Major Beattie, who was in medical charge of the base hospital at Cairo throughout the Egyptian campaign, has written of the work performed by the nursing sisters in terms of the highest commendation. Wherever they went, from base to front, their services were acknowledged by the army doctors to have been the means of saving life, and of lightening the labours and relieving the anxieties of those officers. I trust before long to see the nursing sister a recognised institution in all the larger military hospitals in India.

The nurses, in the first instance at any rate, would have to be got out from home; this would not cost much if Government would grant them passages in troopships. For the work in the military hospitals in this country they would have to be ladies in every sense of the word, otherwise innumerable complications would be sure to arise; and they must have the highest certificates of qualification.

No salary that it is at all likely we could afford to offer would tempt the ordinary run of paid nursing sisters to come out to this country; but there are numbers of devoted women who would come for love of the work and for the sake of the good which they could do, if we could insure them enough to live upon, and their being sent back to England should their health break down. When in Bombay, in March last, I discussed the subject of the possibility of getting ladies to come out for this work with Sister Gertrude Anna, the lady who, with her staff of nursing sisters, has done such good work in the General Hospital in Bombay. She did not think there would be difficulty, and promised the benefit of her experience in selecting efficient nurses, and she would no doubt look after them on first coming out, etc. The sisters would be subject to the War Office regulations for nursing sisters in the military hospitals at home, and would of course be under the medical officer's orders, whose directions they would carry out.

One great difficuly to be contended with is the climate of the plains during the summer months. It is so enervating to the constitution of British women, especially those engaged in the anxious and trying work of nursing, that they could not be expected to retain their health, unless they had homes provided for them in the hills, to which they could resort when not actually engaged in nursing in the military hospitals in the the plains. These homes, I think, could be provided for by private subscription.

What I would propose is, that certain military circles should be defined, near which should be hill stations, or healthy stations, such as Bangalore and Poona, where the sisters would usually reside during the hottest months, and where their services would be available, and a great boon to many, when not required in the military hospitals in the plains. The number of nurses must of course depend upon the extent to which Government would be disposed to assist the movement, and the amount raised by private subscription. We may have to begin in a very small way, but eventually I hope to see homes established at Murree, Kussowlie, Nynee Tal, and Darjeeling, in Bengal; at Bangalore and Wellington, in Madras; and Poona, in Bombay.

In every country except India nursing sisters are to be found in time of war; but to be really useful in India they must gain some experience of the effects of climate, etc., in time of peace. Should war break out, they could be very soon collected and moved to the base of operations, where they would form a valuable staff of nurses, setting free the able-bodied men whose time has hitherto been taken up in looking after the sick and wounded, but whose services would be of so much greater value to the State if employed in the front.

The first step is to get the sanction of Government to the attempt being made to establish a system of female nursing in the military hospitals in India, and a promise of such help as they are disposed to give; the next, to open a subscription fund, which I have every hope will be liberally responded to; for surely no one could refuse to help in such a cause who is ever likely to have friends or relations in the Army in India, exposed in the summer months to the unhealthy climate of the plains, and liable to be struck down at any moment by dangerous illness. Who would be content to think of anyone dear to him lying sick unto death in a military hospital in India, attended doubtless by a kind and clever doctor, but one whose time is fully occupied with numberless other patients, with no one to see that doctor's directions intelligently carried out; no one to minister to the patient's incessant wants, to give him the nourishment on the frequent administration of which his life depends; no one to soothe him with a gentle touch, or calm him with a sympathising word; no one, in fact, near him but "the orderly on duty for the day," who may never have been by a sick bed before, utterly untrained, and ignorant of the requirements of a sick man, and whose every movement and every tone, however kindly meant, is sure to be a source of irritation to the patient, for whom excitement, annoyance, or rough handling meant death?

